# Surface modified niosomal quercetin with cationic lipid: an appropriate drug delivery system against *Pseudomonas aeruginosa* Infections

**DOI:** 10.1038/s41598-024-64416-7

**Published:** 2024-06-11

**Authors:** Jaber Hemmati, Mohsen Chiani, Zahra Chegini, Alexander Seifalian, Mohammad Reza Arabestani

**Affiliations:** 1 Department of Microbiology, School of Medicine, Hamadan University of Medical Sciences, Hamadan, Iran; 2 Student Research Committee, Hamadan University of Medical Sciences, Hamadan, Iran; 3 Department of NanoBiotechnology, Pasteur Institute of Iran, Tehran, Iran; 4 Nanotechnology & Regenerative Medicine Commercialization Centre (NanoRegMed Ltd, Nanoloom Ltd & Liberum Health Ltd), LBIC, University of London, London, UK; 5 Infectious Disease Research Center, Hamadan University of Medical Sciences, Hamadan, Iran

**Keywords:** Niosomal drug delivery system, Stearylamine, Quercetin, *Pseudomonas aeruginosa*, Drug-resistant infection, Biofilm, Nanotechnology, Clinical infection, Microbiology, Molecular biology, Medical research

## Abstract

The Increase in infections caused by resistant strains of *Pseudomonas aeruginosa* poses a formidable challenge to global healthcare systems. *P. aeruginosa* is capable of causing severe human infections across diverse anatomical sites, presenting considerable therapeutic obstacles due to its heightened drug resistance. Niosomal drug delivery systems offer enhanced pharmaceutical potential for loaded contents due to their desirable properties, mainly providing a controlled-release profile. This study aimed to formulate an optimized niosomal drug delivery system incorporating stearylamine (SA) to augment the anti-bacterial and anti-biofilm activities of quercetin (QCT) against both standard and clinical strains of *P. aeruginosa*. QCT-loaded niosome (QCT-niosome) and QCT-loaded SA- niosome (QCT-SA- niosome) were synthesized by the thin-film hydration technique, and their physicochemical characteristics were evaluated by field emission scanning electron microscopy (FE-SEM), zeta potential measurement, entrapment efficacy (EE%), and in vitro release profile. The anti-*P. aeruginosa* activity of synthesized niosomes was assessed using minimum inhibitory and bactericidal concentrations (MICs/MBCs) and compared with free QCT. Additionally, the minimum biofilm inhibitory and eradication concentrations (MBICs/MBECs) were carried out to analyze the ability of QCT-niosome and QCT-SA-niosome against *P. aeruginosa* biofilms. Furthermore, the cytotoxicity assay was conducted on the L929 mouse fibroblasts cell line to evaluate the biocompatibility of the formulated niosomes. FE-SEM analysis revealed that both synthesized niosomal formulations exhibited spherical morphology with different sizes (57.4 nm for QCT-niosome and 178.9 nm for QCT-SA-niosome). The EE% for cationic and standard niosomal formulations was reported at 75.9% and 59.6%, respectively. Both formulations showed an in vitro sustained-release profile, and QCT-SA-niosome exhibited greater stability during a 4-month storage time compared to QCT-niosome. Microbial experiments indicated that both prepared formulations had higher anti-bacterial and anti-biofilm activities than free QCT. Also, the QCT-SA-niosome exhibited greater reductions in MIC, MBC, MBIC, and MBEC values compared to the QCT-niosome at equivalent concentrations. This study supports the potential of QCT-niosome and QCT-SA-niosome as effective agents against *P. aeruginosa* infections, manifesting significant anti-bacterial and anti-biofilm efficacy alongside biocompatibility with L929 cell lines. Furthermore, our results suggest that optimized QCT-niosome with cationic lipids could efficiently target *P. aeruginosa* cells with negligible cytotoxic effect.

## Introduction

*Pseudomonas aeruginosa*, as a primary common nosocomial bacteria, has become a significant challenge for healthcare systems due to its high mortality rate in hospitalized patients^1,2^. The emergence of drug-resistant infections associated with *P. aeruginosa* strains is a significant public health concern, making it challenging to select appropriate treatments^3^. Dissemination of transferable resistance determinants, high ability to biofilm formation, and patient-to-patient transmission are among the main drug-resistance mechanisms in *P. aeruginosa* strains^4^. Also, the inability of conventional antimicrobial approaches to effectively treat resistant *P. aeruginosa* infections leads scientists to find novel therapeutic strategies, particularly vesicular drug delivery systems (VDDSs)^5^.

Recently, VDDSs have offered promising solutions to address the growing threat of bacterial antibiotic resistance, significantly improving patient outcomes in treating related infections^6^. These systems are designed to deliver therapeutic agents specifically to the desired locates, maximizing the therapeutic efficacy and reducing unfavorable side effects. By targeting the bacteria directly, VDDSs could overcome the challenges posed by resistant strains that have developed mechanisms to evade traditional antibiotics^7^. Furthermore, VDDS, by enhancing stability, improving bioavailability, and inhibiting enzymatic degradation, could prolong the drug’s effective dosage at the infected sites. Lipid-based nanocarriers, as one of the approaches of VDDS, can effectively encapsulate broad-spectrum antimicrobial agents, which could be administrated against bacterial infections through drug release in a controlled manner^8^.

Niosomes, as a prominent VDDS, are composed of non-ionic surfactants and cholesterol, which resemble liposomes in structure but offer distinct advantages^5^. These nanoscale vesicles are composed of lipid bilayers and can encapsulate a variety of therapeutic agents, including drugs, peptides, genes, etc., which can turn them into suitable options for different therapeutic purposes^9^. Also, niosomes can encapsulate hydrophilic and lipophilic drugs within their aqueous core and lipid bilayers, respectively^10^. Furthermore, the composition and preparation method of niosomes can be tailored to optimize the drug encapsulation efficiency and its release profile. The versatility of niosomes allows for surface modifications with cationic lipid moieties, such as stearylamine (SA), to achieve a powerful drug delivery^11^.

Quercetin (QCT), 3,3′,4′,5,7-pentahydroxylflavone, is known as a natural flavonoid, which is widely found in various fruits, vegetables, and medicinal herbs including, grapes, onions, tomatoes, tea, fennel leaves, dill, elderberry, cranberry, etc.^12,13^. This natural compound possesses remarkable pharmaceutical properties, including anti-tumor, anticancer, anti-oxidation, anti-inflammatory, anti-viral, and cardiovascular protective^14^. Notably, previous studies on the biomedical properties of QCT proved that it acts as a natural anti-bacterial agent against Gram-positive and Gram-negative bacteria, including *Salmonella enterica*, *Staphylococcus aureus*, *P. aeruginosa*, and *Escherichia coli*^15^.

This study attempts to design a surface-modified niosomal formulation containing QCT, to achieve efficient anti-bacterial activity with reduced cytotoxic effect. By assessing the potential of optimized niosomal formulation with cationic lipids to deliver the effective dosage of the QCT, this research aims to introduce a novel DDS against *P. aeruginosa* infection.

## Material and methods

### Materials

Tween 60 (polyoxyethylene sorbitan monopalmitate), Span 60 (sorbitan monostearate), SA, chloroform, crystal violet, methanol, and all culture media were provided by Merck Company, Germany. QCT was also purchased from Sigma-Aldrich, India, which was prepared in dimethyl sulfoxide as a stock solution (10 mg/mL) for all the experiments. Other materials and reagents were in analytical grade. Spectra/ Por® dialysis membrane (MWCO 12 KDa) was taken from Sigma-Aldrich, U.S.A. *P. aeruginosa* ATCC 27853, as standard strain, provided by the Department of Microbiology, Hamadan University of Medical Sciences, Hamadan, Iran.

### Bacterial isolation

From November of 2023 to January of 2024, a total of 25 clinical strains were collected from patients admitted to Firoozgar Hospital, Tehran, Iran. The isolates were transferred to the laboratory of the Department of Bacteriology, Pasteur Institute of Iran and were diagnosed as *P. aeruginosa* using routine microbiological methods, including Gram stain and biochemical tests. Finally, *P. aeruginosa* strains were stored in trypticase soy broth (TSB) medium at − 20 °C for further investigation^16^.

### Biofilm formation method

The biofilm formation capability of *P. aeruginosa* strains was determined using the microtiter plate (MTP) technique^17,18^. For this purpose, the strains inoculated into 5 ml TSB medium supplemented with 1.5% glucose were incubated overnight at 37 °C. Then, 200 µl of diluted bacterial suspension (1 × 10^6^ CFU ml^−1^) was poured into a 96-well microtiter plate (JetBiofil, Guangzhou, China) and subsequently incubated at 37 °C for 24 h. In the next step, the wells were triplicate washed with 200 μl sterile PBS (pH 7.4), and the formed biofilms were fixed by absolute methanol for 15 min. Afterward, the biofilms were stained with 200 μl of crystal violet solution (1.5%w/v) and were solubilized with 150 μl of acetic acid solution (33%v/v). Finally, wells’ optical densities (ODs) were evaluated using a microplate ELISA reader (Biotek, USA) at 570 nm. The biofilm formation patterns were classified into weak, moderate, and strong groups by comparing the ODs of wells with the cut-off OD value (ODc). The ODc was obtained as three standard deviations (SD) above the mean absorbance of the negative control. Notably, all assays were carried out in triplicate, and *P. aeruginosa* ATCC 27853, as a strong biofilm-producing strain, and uninoculated TSB medium were considered positive and negative controls, respectively^19^.

### Preparation of niosomal formulations

The niosomal formulation was prepared using the thin-film hydration method^20^. Firstly, a specific amount of Span 60, cholesterol, and Tween 60 with a molar ratio of 2:2:1 (227.8 mg: 347.0 mg: 204.5 mg) was dissolved in 20 ml organic solvent (chloroform/methanol 2:1 v/v) using a magnetic stirrer (150 rpm, 50 min, 25 °C) for obtaining a homogenized solution. Then, the organic solvent was evaporated using rotary evaporation (WB Eco Laborota 4000 Model, Heidolph Instruments, Germany) under vacuum at 60 °C for 45 min. The remaining solvent was removed by purging the nitrogen gas, and the dried lipid was hydrated in 20 ml of 100 mM saline (PBS, pH 7.4) containing QCT for 45 min. The molar ratio of lipid to the drug was 20:1. Finally, the synthesized niosome was sonicated for 10 min by a probe sonicator (Hielscher up50H ultrasonic processor, Germany). QCT-SA-niosome was synthesized with the same procedure with the addition of SA, which its molar ratio to lipid was considered 25:2. To ensure complete dissolution and uniformity of niosomes, the prepared formulations were visually observed for any particulates or aggregates and were kept at 4 °C for further experiment.

### Characterization of the prepared niosomes

#### Particle morphology

The morphology, size, and uniformity of the prepared niosomes were assessed by field emission scanning electron microscopy (FE-SEM) (Hitachi S4160, Japan). Briefly, one drop of the niosomal suspension diluted 1:100 in deionized water was mounted on the plate base and coated with a conductive gold layer. Notably, the taken pictures were analyzed using ImageJ software (bundled with Java 1.8.0_172).

#### Determination of particle size and surface zeta potential

Particle size, zeta potential, and poly dispersity index (PDI) of synthesized niosomes were evaluated by dynamic light scattering (DLS) method using a zetasizer instrument (HORIBA SZ-100, Japan) at 633 nm. For this purpose, the samples were diluted 1:100 with PBS (pH 7.4) and analyzed in a polystyrene cuvette at the same concentration, pH, and temperature (0.1 mg/ml, pH 7.4, 25 °C). Notably, the analyses were repeated three times, and the average of results was determined.

#### Assessment of entrapped drug in niosomal formulations

The entrapment efficiency (EE%) of formulated niosomes was determined using the ultra-centrifugation method^21^. In Brief, 1 ml of niosomal formulations containing QCT was centrifuged for 15 min at 14,000 g at 4 °C in an Amicon ultra centrifugal filter (molecular weight cut-off of 50 kDa, Merck Millipore Ltd.). The amount of QCT in supernatant solution (un-entrapped) was measured by UV spectrophotometry (Jasco V-530, Japan) at 372 nm^22^. A calibration curve was prepared by different concentrations of QCT (100–800 µg/mL) in methanol to estimate the amount of QCT. Finally, the EE% of QCT in niosomal formulation was reported using the following equation: Entrapment Efficiency (EE)% = [(A − B)/A] × 100.

Whereas A is the amounts of QCT fed initially into the niosomal formulation, and B is the amount of free QCT in the supernatant solution.

#### In vitro drug release analysis of niosomal formulation

The in vitro study of drug release from QCT-niosome and QCT-SA-niosome formulations was carried out using dialysis methods^23^. Firstly, 1 ml of free and encapsulated drugs were added into a dialysis bag immersed in 25 ml recipient medium (PBS, pH = 7.4, 5 mM) and magnetically stirred at 100 rpm at 37 °C. At intervals of 1, 2, 4, 8, and 24 h, 1 ml of the recipient medium was aliquoted and spectrophotometrically evaluated. Finally, the OD of samples was estimated based on the standard curve equation, and the concentrations of the released QCT at each time interval were estimated. Notably, the withdrawn samples were replaced with a fresh medium at 37 °C.

#### Stability studies

The stability of the prepared formulation was accomplished by measuring the vesicle size, PDI, and EE% for both QCT-niosome and QCT-SA-niosome formulations every month for a 4-month storage period at 4 °C and 25 °C.

#### Cytotoxicity determination of niosomal formulations

For biocompatibility analysis of synthesized niosomes, the cytotoxicity of both niosomal formulations was determined using MTT (dimethylthiazol-2-yl)-2, 5-diphenyl-tetrazolium bromide) method^24^. In Brief, the L929 mouse fibroblast cell line (which was provided by the cell bank of Pasture Institute of Iran, Tehran, Iran) was cultured into a polystyrene 96-well microtiter plate and incubated under sterile conditions with 5% CO_2_ at 37 °C. Afterward, the increasing concentrations of niosomal formulations were added to L929 cells, and the microtiter plate was incubated overnight at 37 °C. In the next step, the wells were resuspended in 15 µl of MTT solution (5 mg/ml) and incubated at 37 °C for 4 h. Finally, 100 µl of dimethyl sulfoxide (DMSO) solution was added into each well, and the ODs were calculated at 570 nm wavelength using a microplate ELISA reader (AccuReader, Metertech, Taiwan). Notably, the cell viability % was determined using the following formula: Cell viability (%) = (OD sample / OD control) × 100.

### Anti-bacterial and anti-biofilm analysis

#### Minimum inhibitory and bactericidal concentrations (MICs/MBCs)

Minimum inhibitory concentrations (MICs) of free QCT, QCT-niosome, and QCT-SA-niosome against *P. aeruginosa* isolates were examined using the approved CLSI broth microdilution assay^25^. For this purpose, Mueller Hinton broth (MHB) containing serial dilutions of samples was added to a sterile 96-well microtiter plate. In the next step, 0.5 McFarland suspensions (1.5 × 10^8^ CFU/ml) of *P. aeruginosa* isolates were added into wells, and the plates were incubated overnight at 37 °C. The minimum sample concentrations inhibiting visible bacterial growth were determined as MICs. Also, MBCs were determined as the lowest concentrations resulting no-growth (> 99%) after 24 h incubation on Mueller–Hinton agar (MHA) at 37 °C. All assays were performed in triplicate, and *P. aeruginosa* ATCC 27853 and uninoculated MHB medium were considered positive and negative controls, respectively.

#### Well diffusion

The anti-bacterial activities of free QCT, QCT-niosome, and QCT-SA-niosome were examined against *P. aeruginosa* strains according to the agar well diffusion method^21^. Firstly, the standard suspensions of selected bacteria were cultured on MHA medium, and the wells with a diameter of 10 mm were created in the plates using a sterile gel puncture. Then, the serial concentrations of samples were added to the wells, and the plates were incubated at 37 °C for 24 h. Finally, the inhibition zones were measured and compared with the diameters of the control well. Notably, gentamicin disk (10 µg) (MAST, UK) and distilled water were considered positive and negative controls, respectively.

#### Biofilm formation analysis

In order to examine the anti-biofilm effect of QCT-niosome and QCT-SA-niosome in comparison to free QCT, the MTP was carried out. For this purpose, 200 μl of 10^6^ CFU/ml suspension of *P. aeruginosa* strains diluted with TSB medium supplemented with 1.5% glucose was added into a polystyrene 96-well microtiter plate. Subsequently, the strains were treated with a sub-MIC concentration of samples and incubated at 37 °C for 24 h. Afterward, the wells were rinsed with sterile PBS (pH 7.4) in triplicate and fixed for 15 min by absolute methanol solution (99.8%). Finally, the formed biofilms were stained with crystal violet solution (1.5%w/v), and the ODs of wells were evaluated in triplicate at 570 nm^26^. Notably, *P. aeruginosa* ATCC 27853 and uninoculated TSB medium were considered positive and negative controls, respectively.

#### Biofilm eradication analysis

Minimal biofilm eradication concentrations (MBECs) were carried out to assess the ability of free QCT, QCT-niosome, and QCT-SA-niosome against *P. aeruginosa* strains. As previously mentioned, the bacterial strains were allowed to form 1- and 3-day-old biofilms. Afterward, the formed biofilms were treated with a sub-MIC concentration of prepared niosomal formulations and incubated at 37 °C for 24 h. Subsequently, the wells’ contents were inoculated on MHA medium for 48 h at 37 °C, and the MBECs were determined as the lowest concentration killing 100% of the embedded bacteria^27^. Notably, the uninoculated TSB medium and *P. aeruginosa* ATCC 27853 were considered negative and positive controls, respectively.

### Statistical analysis

The statistical analysis between investigated parameters was examined using the t-test. Also, a statistically significant difference was considered at *P*-value less than 0.05 for all comparisons. Notably, graphs were generated using GraphPad Prism version 9.0 software.

### Ethics approval and consent to participate

This study was approved by the Ethics Committee of Hamadan University of Medical Sciences, Institutional Review Board (IR.UMSHA.REC.1402. 432). The experiments in our study were conducted in accordance with relevant guidelines and regulations, as well as the Declaration of Helsinki. Informed consent was obtained from all patients and the parents or legal guardians of children participating in the study.

## Results

### Bacterial isolation and biofilm formation

**Figure 1:**
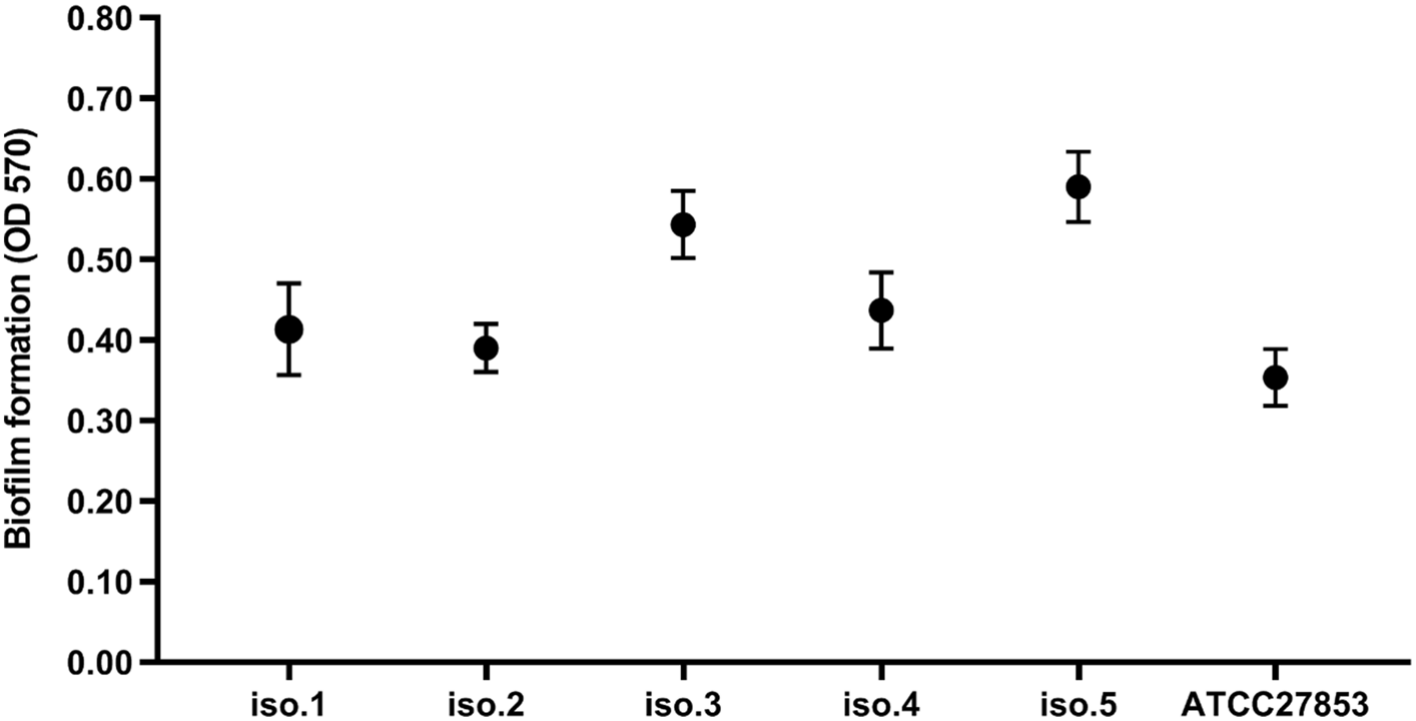
Biofilm formation values of *P. aeruginosa* strains obtained by the microtiter plate assay (mean ± SD, n = 3).

A total of 5 *P. aeruginosa* strains were recovered from 25 clinical samples. According to the results of the MTP method, all *P. aeruginosa* strains were determined as strong biofilm-formers (Fig. 1).

### Physicochemical characterization of prepared niosomes

#### Morphology, size, PDI, and zeta potential

**Figure 2:**
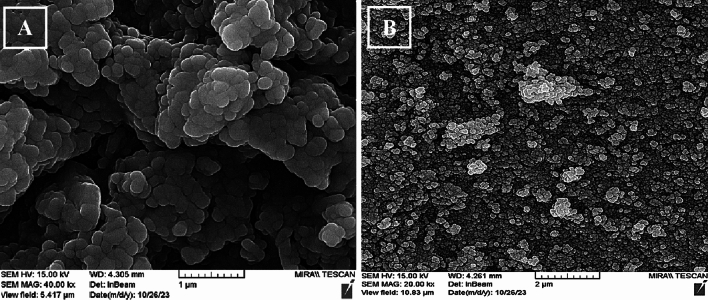
Spherical morphology of QCT-SA-niosome (**A**) and QCT-niosome (**B**) according to the field emission scanning electron microscopy (FE-SEM).

**Figure 3:**
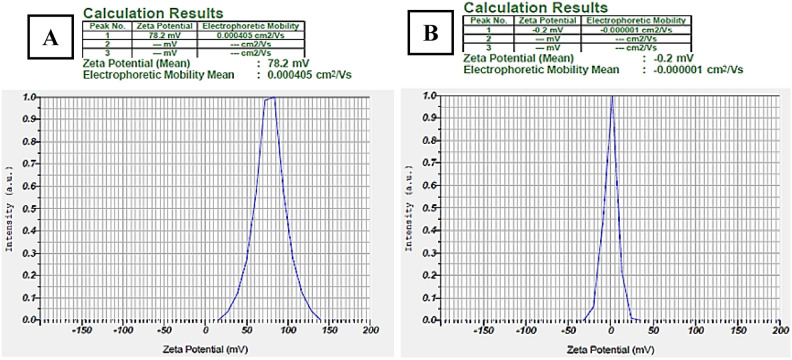
Zeta surface potential of QCT-SA-niosome (**A**) and QCT-niosome (**B**) obtained from dynamic light scattering (DLS) analysis.

Based on the micrograph obtained from FE-SEM, both niosomal formulations (QCT-niosome and QCT-SA-niosome) were uniform and spherically shaped (Fig. 2). The average size of niosome particles measured by FE-SEM was 178.9 nm for QCT-SA-niosome and 57.4 nm for QCT-niosome. The diameter measured by the zetasizer was 194.1 nm for SA-niosomal QCT and 68.9 nm for niosomal QCT. Also, the size distributions (PDI) of QCT-niosome and QCT-SA-niosome were reported at 0.152 and 0.219, respectively, indicating homogenic dispersion for both formulations. The surface charges of QCT-niosome and QCT-SA-niosome were also reported at − 0.2 mV and + 78.2 mV, respectively (Fig. 3).

#### Entrapment efficiency analysis

Encapsulation into VDDS could be known to enhance drug pharmaceutical activities, considered an essential factor for applying of niosomal delivery system in medical applications^28^. In our research, the amount of encapsulated drug in standard niosomal suspension was measured at 59.6%, while the EE% rate of SA-niosomal formulation was reported at 75.9%. The comparison of these results indicated that a high yield of drug EE% was obtained by incorporating SA into niosomal formulation.

#### Drug release profile

**Figure 4:**
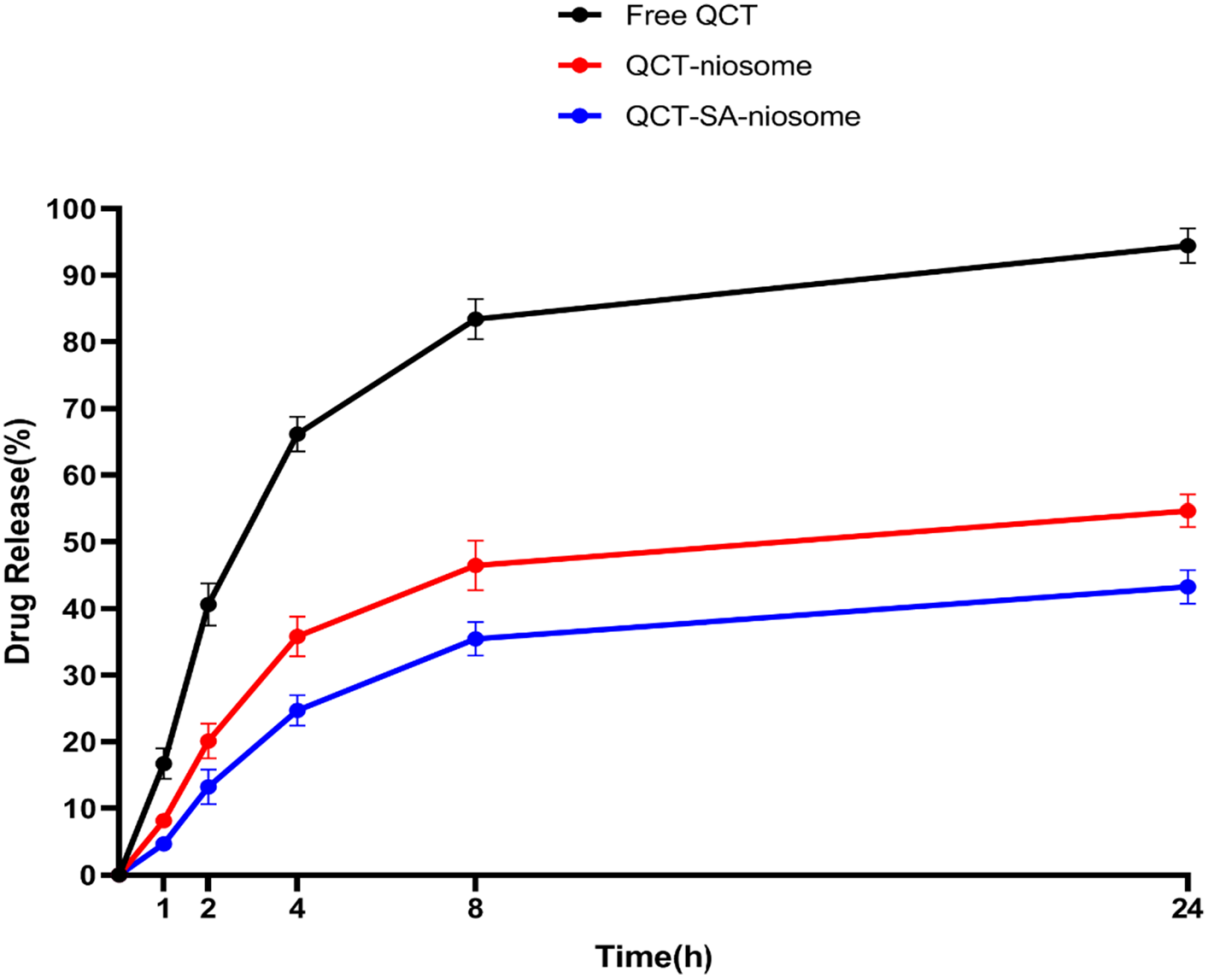
Comparative drug release profile of free QCT, QCT-niosome, and QCT-SA-niosome in recipient medium (PBS, pH = 7.4, 5 mM, 37 °C) (mean ± SD, n = 3).

Figure 4 compares drug release rates from free QCT, QCT-niosome, and QCT-SA-niosome using the dialysis bag method. As shown, around 70% of the drug was released within four hours from free formulation, while only 36% and 25% of the encapsulated drug was released from QCT-niosome and QCT-SA-niosome formulations during the same time, respectively. Also, in the first 24 h, approximately 100% of the drug was released from free formulation, while the highest rates of drug release from QCT-niosome and QCT-SA-niosome formulations were almost 55% and 43%, respectively. The comparison results showed that the considerable drug release could be hindered by entrapping into niosomal formulation. Furthermore, the SA-niosomal formulation had a better drug release profile than the standard formulation, which could be considered in preparing niosomal DDS with a sustaining release profile.

#### Physical stability study of synthesized niosomal formulations

**Figure 5:**
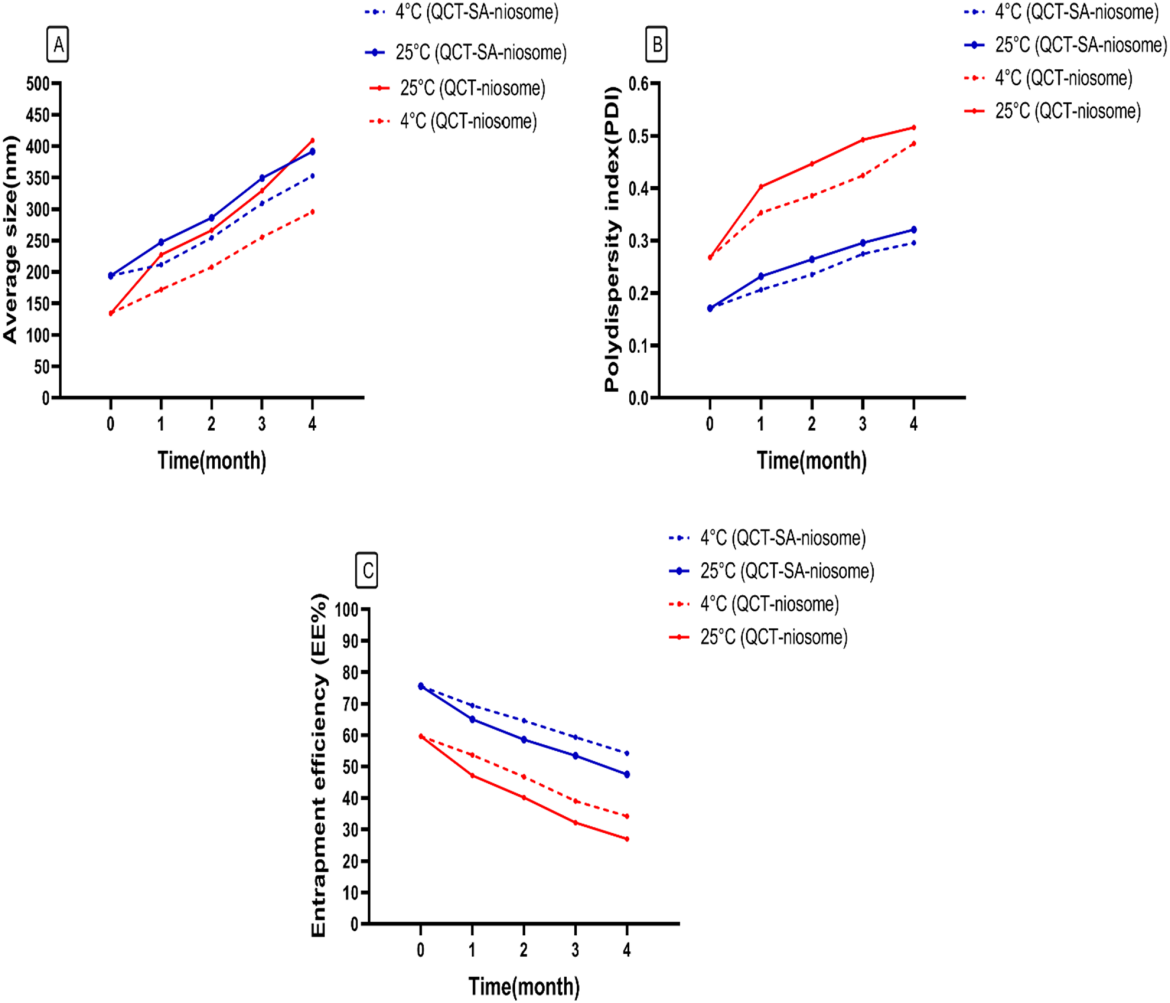
Stability of QCT-niosome and QCT-SA-niosome formulations stored during a 4-month storage time at 4 °C and 25 °C. (**A**) particle size, (**B**) polydispersity index (PDI), (**C**) entrapment efficiency %.

The physical stability of formulated niosomes was assessed by determining size, PDI, and EE% during four months of storage at 4 °C and 25 °C (Fig. 5). These findings showed that all examined parameters changed slower at 4 °C than 25 °C. Also, the results revealed the more physical stability of formulated QCT-SA-niosome compared with QCT-niosome, suggesting that cationic lipid had an effective role in niosomal stability.

#### Niosomal cytotoxicity

**Figure 6:**
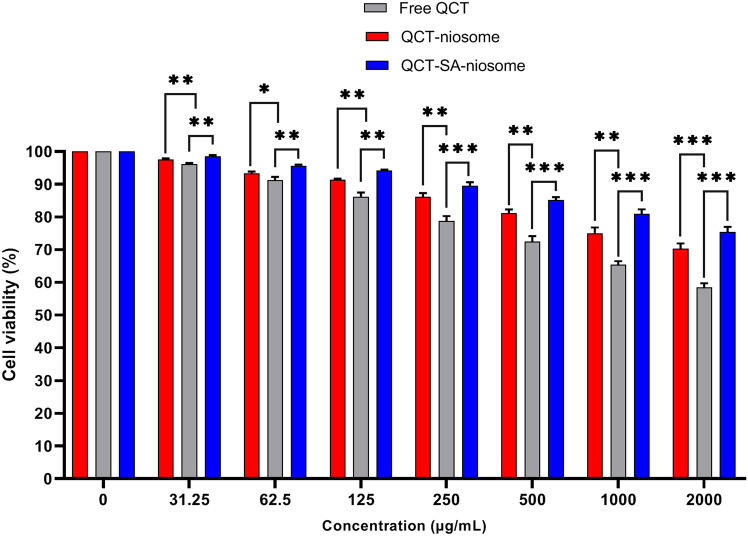
Cell viability of free QCT, QCT-niosome, and QCT-SA-niosome on L929 cell line (mean ± SD, n = 3, ns: not significant, *: *P* < 0.05, **: *P* < 0.01, ***: *P* < 0.001).

The viability of different concentrations of free QCT, QCT-niosome, and QCT-SA-niosome was investigated on the L929 cell line (Fig. 6). Our findings revealed the significantly lower cytotoxicity effect of QCT-niosome compared to free QCT. Also, the cytotoxicity of QCT-SA-niosome was less than QCT-niosome at the same concentrations, indicating that the presence of SA in niosomal component could effectively reduce the side effects of the loaded drug. Notably, the cytotoxicity of the blank niosome was examined, and no toxicity was shown against L929 cells.

### Assessment of anti-bacterial and anti-biofilm ability

#### MIC and MBC

**Figure 7:**
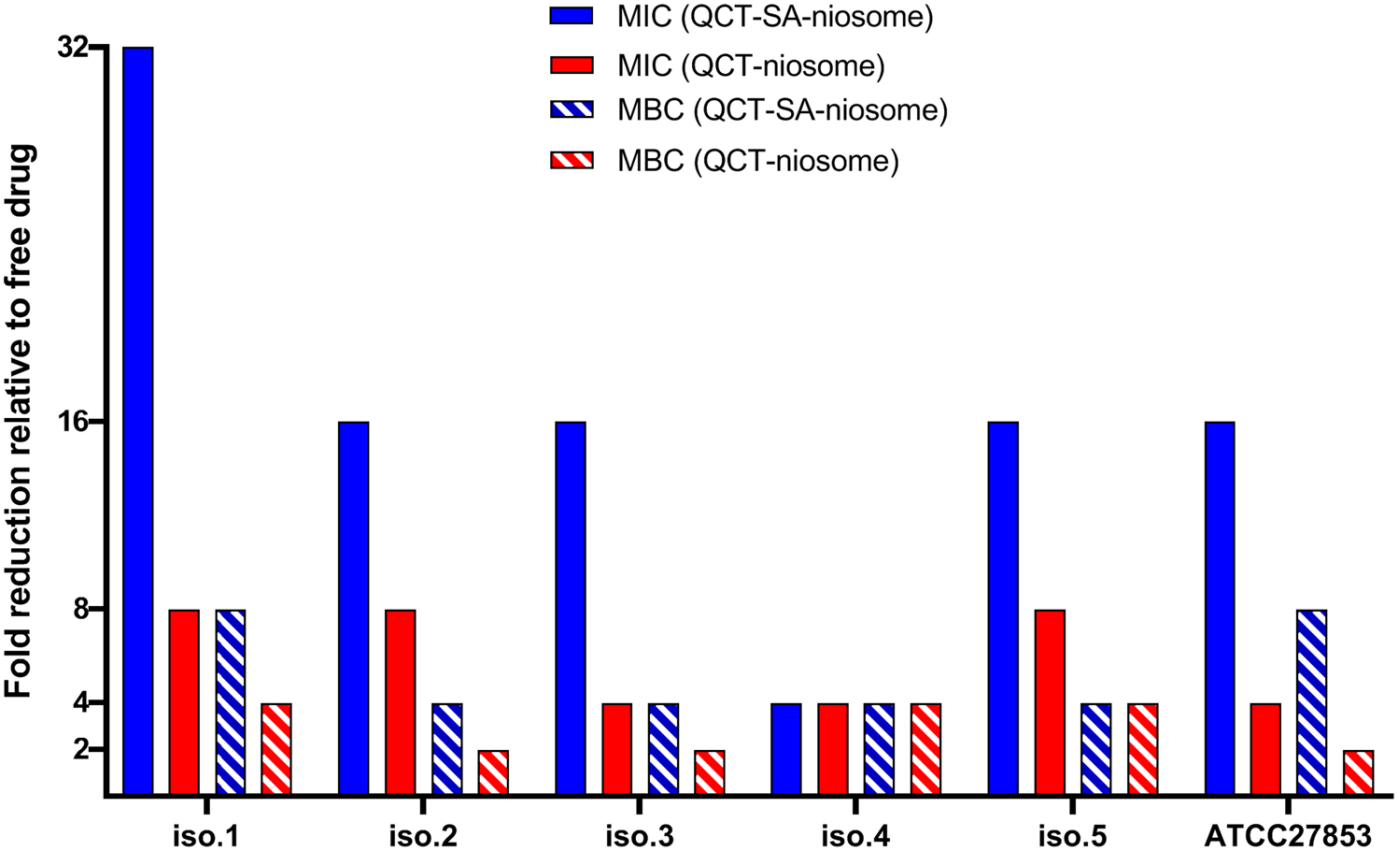
Comparison of anti-bacterial activity of QCT-niosome and QCT-SA-niosome with free QCT against *P. aeruginosa* strains.

The MICs and MBCs of QCT-niosome and QCT-SA-niosome were assessed against *P. aeruginosa* isolates and compared to the free QCT (Fig. 7). According to our results, QCT-niosome and QCT-SA-niosome had a significant inhibitory effect against all *P. aeruginosa* strains, decreasing the MIC values of free QCT by 4–8 and 4–32-fold, respectively. Also, the niosomal drug exhibited high bactericidal ability, and the MBC values of the QCT-niosome and QCT-SA-niosome were lower than the free drug by 2–4 and 4–8, respectively. Notably, the anti-bacterial activity of the blank formulation was also measured, but no anti-bacterial effect against *P. aeruginosa* isolates was shown.

#### Well diffusion

The results of the well diffusion method exhibited that the inhibition zone of QCT-niosome and QCT-SA-niosome were significant compared with free QCT against all *P. aeruginosa* strains. Moreover, the growth inhibition zones were dose-dependent as they improved with increasing the concentration of both niosomal formulations (Table 1).

**Table 1:** The growth inhibition zones (in mm) of free QCT, QCT-niosome, and QCT-SA- niosome against *P. aeruginosa* isolates.

	Bacterial isolates					
	iso. 1	iso. 2	iso. 3	iso. 4	iso. 5	ATCC 27853
125 µg/mL free QCT/QCT-niosome/QCT-SA-niosome	2.31/4.23/6.32	1.69/3.86/5.37	2.36/3.98/5.12	1.54/3.21/4.55	1.96/3.12/4.95	1.25/2.32/2.84
250 µg/mL free QCT/QCT-niosome/QCT-SA-niosome	3.27/5.62/7.59	2.47/4.75/6.80	3.55/5.27/6.70	2.31/4.55/6.35	2.65/4.51/6.32	2.13/3.41/4.60
500 µg/mL free QCT/QCT-niosome/QCT-SA-niosome	4.25/6.38/9.65	3.84/6.32/8.37	4.32/6.65/8.57	3.50/6.49/8.15	3.31/7.05/8.95	3.87/5.41/6.82
1000 µg/mL free QCT/QCT-niosome/QCT-SA-niosome	5.12/7.90/12.25	5.36/8.65/10.34	5.51/8.74/10.95	5.32/8.85/11.65	4.62/9.84/12.36	6.31/7.29/9.34

#### Biofilm formation

**Figure 8:**
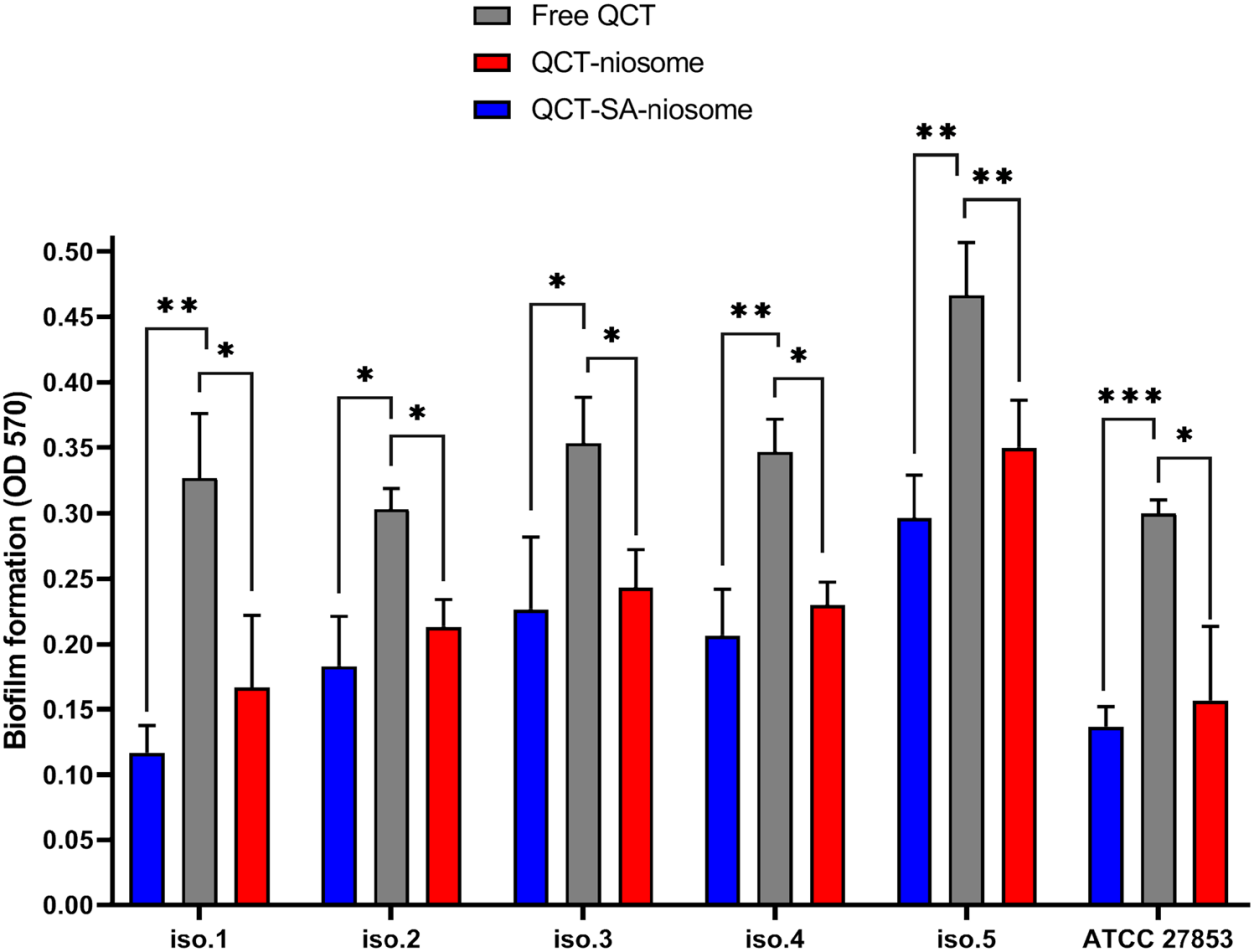
Anti-biofilm activity of free QCT, QCT-niosome, and QCT-SA-niosome against *P. aeruginosa* strains (mean ± SD, n = 3, *: *P* < 0.05, **: *P* < 0.01, ***: *P* < 0.001).

The inhibitory efficacy of synthesized niosomes on *P. aeruginosa* biofilm formation was evaluated and compared with the free drug (Fig. 8). The results of the MTP assay revealed that treatment of *P. aeruginosa* isolates with both QCT-niosome and QCT-SA-niosome formulations significantly inhibited biofilm formation compared to the free QCT. Also, our results revealed that QCT-SA-niosomal formulation had a higher inhibitory activity on biofilm formation compared with QCT-niosome. Notably, the anti-biofilm effect of blank niosome was measured, which had no inhibitory efficacy on biofilm formation against all *P. aeruginosa* isolates.

#### Biofilm eradication

**Figure 9:**
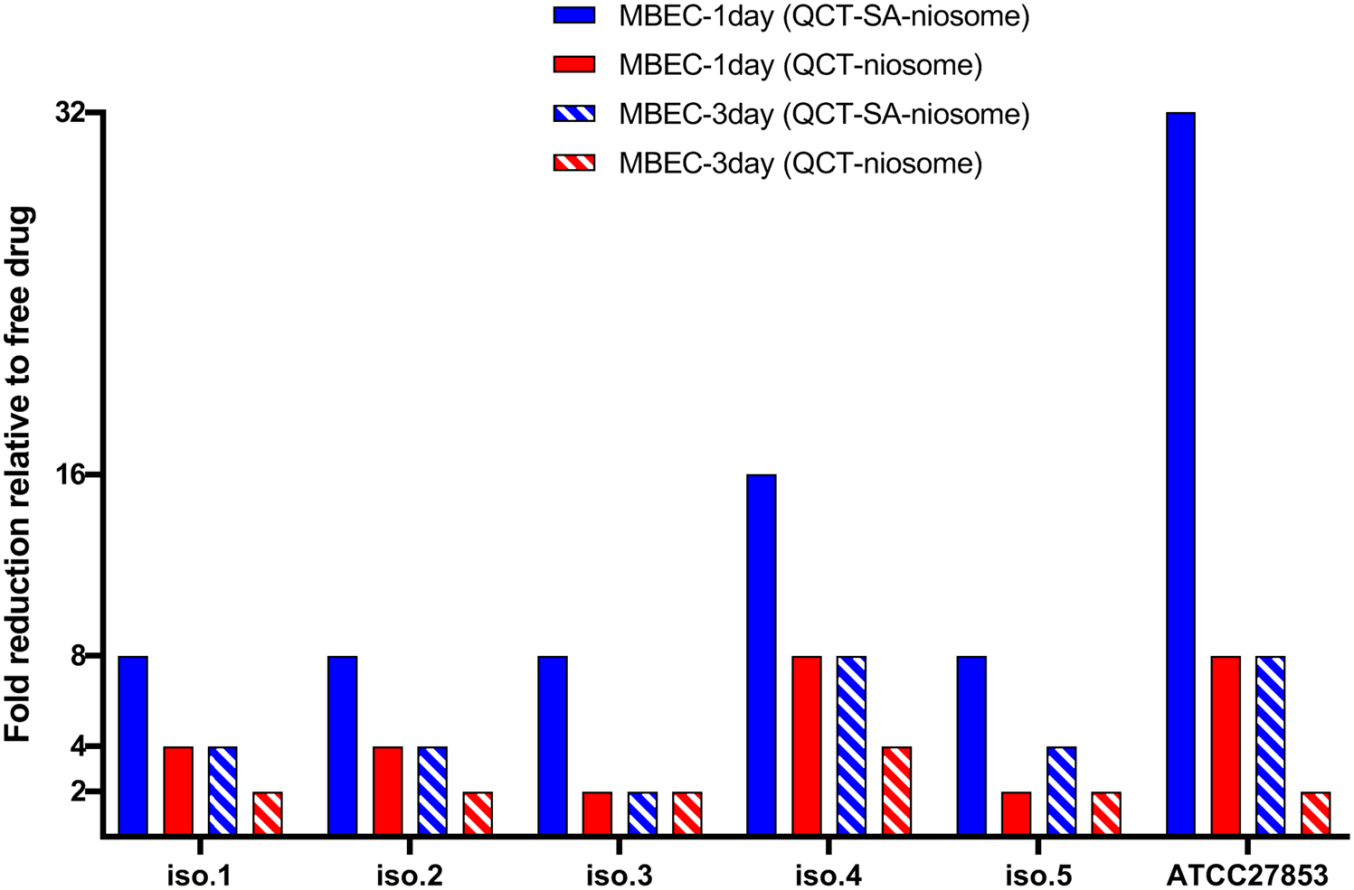
Comparison of minimum biofilm eradication concentrations (MBECs) of QCT-niosome and QCT-SA-niosome with free QCT against *P. aeruginosa* strains.

The anti-biofilm efficacy of QCT-niosome and QCT-SA-niosome against *P. aeruginosa* strains was assessed by determining MBECs and compared with free QCT (Fig. 9). Based on our results, QCT-SA-niosome and QCT-niosome formulations decreased the 1-day-MBEC values of free QCT by 8–32-fold and 2–8, respectively, against all *P. aeruginosa* isolates. Also, the results of MBEC revealed that both QCT-niosome and QCT-SA-niosome eradicated 3-day-old *P. aeruginosa* biofilms at lower concentrations than non-encapsulated drug. Notably, the anti-biofilm efficacy of blank niosomes was determined, which failed to eradicate *P. aeruginosa* biofilms at the same concentrations of prepared niosomal formulations.

## Discussion

*P. aeruginosa* is known as a prominent complicated bacterial pathogens, employing various resistance mechanisms mainly based on the presence of efflux pump system, the limited permeability of outer membrane, and high ability to biofilm formation^29^. The fusional interactions between the bacterial surface and VDDS could be promising an effective approach in dealing with drug resistance^5^. In this regard, Satish et al. exhibited a significant increase in the anti-bacterial efficacy of fluoroquinolones encapsulated in niosomal system against both Gram-positive and Gram-negative bacteria^30^. According to their results, the niosomal formulations of four fluoroquinolone drugs (ciprofloxacin, gatifloxacin, levofloxacin, norfloxacin) produced at least a twofold reduction in MICs against *P. aeruginosa* and *E. coli* isolates, and at least a fourfold reduction in MICs against *S. aureus* isolates (*P*-value < 0.0001). Also, another study demonstrated the improved anti-bacterial activity of niosomal DDS against 12 *P. aeruginosa* strains, where tobramycin-loaded niosomes resulted in a considerable reduction in both MIC and MBC values compared to free drug in the range of 4–32-fold^31^. Additionally, Mansouri et al.^32^ showed that the streptomycin sulfate–loaded niosomes had a higher anti-bacterial effect against *P. aeruginosa* strains than free drug, which reduced the MIC and MBC values by fourfold and eightfold, respectively. In line with the mentioned studies, we also found that drug-encapsulated niosomes had more anti-bacterial potential against *P. aeruginosa* strains than free ones. So, the MICs of incorporated QCT were 2–32-fold lower than the free QCT. Furthermore, our results exhibited that niosomal formulation had high bactericidal efficacy against all *P. aeruginosa* strains, and the MBC concentrations of QCT-niosome were 2-fourfold lower than free drug. These findings suggest that a lower concentration of niosomal drug was required to inhibit bacterial growth, and were approved by the well diffusion method. According to our results, the inhibition zones of niosomal QCT against *P. aeruginosa* strains were greater than the free QCT in all concentrations (125, 250, 500, and 1000 µg/mL). Moreover, the diameters of the growth inhibition zone were dose-dependent as they enhanced with increasing the concentration of niosomal formulations. The results of the well diffusion method were also approved by Heidari et al.’s study^21^, where tannic acid-loaded niosomes had a greater growth inhibition zone against *P. aeruginosa* compared with free tannic acid. Furthermore, the analysis of well diffusion’s results reported by Akbarzadeh et al.^33^ indicated that encapsulation of doxycycline into niosome improved its anti-bacterial efficacy against different Gram-positive and Gram-negative bacteria, including *P. aeruginosa* strains. In the current investigation, the inhibitory ability of niosomal entrapment on *P. aeruginosa* biofilms was also evaluated, and it was proven that niosomal formulation had a high potential for dealing with biofilm-forming *P. aeruginosa* strains. Also, the MTP results showed that the biofilm formation ability among *P. aeruginosa* strains was significantly lower in the case of niosomal QCT as compared to its free peer (*P*-value < 0.05). In addition, the anti-biofilm efficacy of prepared formulations was investigated by MBEC assay, which both QCT-niosome and QCT-SA-niosome significantly eradicated 1- and 3-day-old *P. aeruginosa* biofilms compared to the free QCT (*P*-value < 0.001). In line with our research, the results of the study^21^ revealed that the inhibitory ability of free drug on *P. aeruginosa* biofilms was significantly increased through encapsulating into niosomal formulation (*P*-value < 0.001). In this regard, Abdelaziz et al.^34^ showed that niosomal DDS could be developed for suppressing *P. aeruginosa* attachment to the abiotic surfaces, where SEM micrograph approved that niosome containing norfloxacin enhanced drug release to the bacterial cell membranes. Also, the results of another study^32^ showed the anti-biofilm effect of the niosomal encapsulation against *P. aeruginosa*, which could significantly improve the inhibitory activity of free drug on biofilm formation (*P*-value < 0.001). In addition, Mahdiun et al.^35^ showed that simultaneously incorporating tobramycin and bismuth-ethanedithiol into niosomal formulation decreased the rate of biofilm formation in *P. aeruginosa* strains by 80%. In addition, Hedayati et al.^36^ found that niosomes significantly increased (*P*-value < 0.001) the anti-biofilm activity of loaded contents, which could be a suitable candidate against drug-resistant *P. aeruginosa* isolates. By review of mentioned studies, it was concluded that niosomes could be developed as a powerful DDS against bacterial-resistant infections by targeting the effective drug dosage at the infected sites. It could be discussed that niosomes could distribute loaded contents inside the cytoplasmic space by interacting with the membrane phospholipid. The interaction between niosomes and bacterial outer membrane is mediated by niosomes’ fusogenic properties, leading to the diffusion of loaded drugs into Gram-negative subcellular space^30,37^. On the other hand, niosomes could prolong drug accessibility to embedded cells by facilitating drug release to biofilm space, efficiently inhibiting of bacterial biofilm formation^38^. Furthermore, niosome nanoparticles could act as a physical barrier and compete with biofilm-producing bacteria for surface adhesion, which could prevent biofilm formation by decreasing bacterial attachment^39–41^.

Recently, VDDS has been gaining attention in nanomedicine, providing astounding applications for conventional drug dosages. The efficacy and functionality of VDDS can be further refined and improved through surface modification. It is proven that drug delivery can become a better targeted process through surface conjugating VDDS with various moieties, increasing the therapeutic efficacy of loaded contents^42^. According to our microbial experiments, niosomal surface modification could increase the pharmaceutical potential of synthesized niosomes, which could be provided through altering niosomal composition with a cationic lipid (SA). These results indicated the anti-bacterial and anti-biofilm abilities of cationic niosomal QCT against both clinical and standard *P. aeruginosa* isolates. The current study showed that QCT-SA-niosome increased the anti-bacterial potential with a reduction in MIC and MBC values of QCT-niosome by 2–fourfold against 80% of tested isolates. Also, the results of the anti-biofilm analysis showed that QCT-SA-niosome had lower MBEC-1 day values compared to QCT-niosome against all *P. aeruginosa* isolates (*P*-value < 0.001). However, the enhanced anti-biofilm effects of QCT-SA-niosome compared to QCT-niosome were not shown against all 3-day-old biofilms. The results of a study determined that preparation of niosomal norfloxacin with cationic agents significantly reduced the MIC and MBC values of free drug by 2–64-fold against *P. aeruginosa* strains^34^. According to their results, a significant anti-biofilm activity was also found against *P. aeruginosa* biofilms through incorporation of positively charged agents into niosomal formulation (*P*-value < 0.05). Also, another conducted study revealed that of niosomal optimization through surface modification with PEGylatation improved the anti-bacterial and anti-biofilm activities of vancomycin-encapsulated niosomes against *S. aureus* strains (*P*-value < 0.05)^43^. The confirmatory results were also reported in the published studies on liposomal formulations. In this regard, cationic liposomes containing aminoglycosides had a greater efficacy on *P. aeruginosa* than standard liposomal formulation^44^. In another study, encapsulation of antibiotics in a fusogenic liposome (with more positive surface charged) was exhibited to reduce the MIC values by 2–fourfold against *P. aeruginosa* compared to the corresponding standard liposomal formulation^45^. Also, another study demonstrated a powerful fusion between Fluidosomes® (liposomal formulation consisting of cationic lipid) and *P. aeruginosa* cells, where tobramycin-loaded Fluidosomes®, in a sub-MIC concentration, found a significant reduction in the bacterial counts compared to the standard liposomal formulation (*P*-value < 0.001)^46^. However, in another study, Fluidosomes® containing meropenem had 4–16 times lower MICs for both clinical and standard isolates than did the free meropenem^47^. In total, the review of studies indicates that drug delivery would be a much more targeted process through conjugating with cationic moieties. It is hypothesized that positively charged vesicles will undergo prolonged and strongly electrostatic interaction/fusion with the negatively charged bacterial cell membrane and biofilms. This superiority is supported by another hypothesis that the pharmaceutics index of antimicrobial agent would be increased by higher encapsulation capacity for the cationic vesicles^48^. It should be considered that most of the research dealing with drugs-encapsulated niosomes concentrated on enhancing the delivery of drugs through different administration routes, with no highlighting of the enhanced antimicrobial activity through surface modification of formulated niosomes^28,48,49^. Also, this hypothesis should be considered that the encapsulated drugs probably interact with niosomal/liposomal lipids, which could inhibit VDDS fusion process with bacterial membrane^47^. Therefore, further studies should be conducted to investigate the exact mechanism of VDDS on anti-bacterial and anti-biofilm of loaded contents and the effect of surface modifications on improving these parameters.

The therapeutic efficacy of VDDS encapsulating different antimicrobial agents depends on successfully designing a formulation with sustained-drug release profile. According to our study, the cationic niosomal formulation exhibited a more sustained-release profile than the standard niosomal formulation. As shown in Fig. 4, the rates of drug release from QCT-SA-niosome were 3.5–11.4% less than QCT-SA-niosome at different time intervals. In addition, the amounts of drug leakage within 8 h from cationic and standard niosomal formulations were around 35% and 45%, respectively. Also, it was demonstrated that around 43% of QCT was released from cationic formulation during 24 h, but the rate of drug release from standard formulation at the same time was around 55%. In agreement with our findings, the results of Webb et al.’s study indicated that SA effectively decreases the drug leakage from liposomes and could be a valuable component in liposomal formulations^50^. According to their release study, the leakage of verapamil from a liposomal formulation containing SA was 40% slower than standard formulation during 24 h. Also, another study suggested that the cumulative drug release process can be significantly inhibited by using cationic liposomes (SA formulation) with a sustained-release effect^51^. This study found that the release rate of cationic lipid-free formulation was 97.82% and was ended in 8 h, but the cumulative release of cationic formulation reached only 73.94% within the same duration. However, in another study, the in vitro release rates from SA-niosomal formulation were 81.8% and 91.9% during 8 and 24 h, respectively^52^. The rationale for the mentioned results was that the permeability of encapsulated drugs could be effectively decreased if the inner monolayer of the niosomes/liposomes possessed a positive surface potential. This issue can be explained by the fact that the drug retention characteristics are directly proportional to its concentration at the membrane surface. So, the charge repulsion due to the presence of SA reduces the local concentration of loaded contents adjacent to the membrane surface, efficiently decreasing the drug concentration gradients across the membrane^53^. Taken in sum, these studies show that SA could reduce the leakage of incorporated drugs from niosomes/liposomes, and may prove to be a valuable ingredient in their formulations. However, more investigation is needed for the exact effect of niosomal surface modification on drug release profile, which could be supported by further kinetic analysis.

In the current research, a stability study for a 4-month period was carried out to compare the ability of both prepared niosomal formulations (QCT-niosome and QCT-SA-niosome) to maintain their physicochemical characteristics. The physical appearance of both cationic and standard formulations was visualized unchanged during four months, and neither flocculation nor sedimentation were seen. Also, the results obtained from the stability study indicated that refrigerated niosomal formulation (4 °C) had a slower change in size, PDI, and EE% than that stored at room temperature (25 °C). The increased leakage of the entrapped drug from the vesicular structure at 25 °C can be justified in this way: the increased temperature induced energy-dependent fusion of niosomes by increasing its Brownian motion; consequently, the phospholipid becoming more fluid and flexible, facilitating the leakage of entrapped drug. Also, the stability analyses declared that during a 4-month storage time, both formulations maintained in the acceptable ranges with a significant change in particle diameter, size distribution (PDI), and EE% (Fig. 5)^54,55^. In addition, the investigated stability parameters (size, PDI, EE%) in the cationic niosomes had fewer changes than the standard formulation, indicating the effective role of SA in niosomal stability. Furthermore, comparing our results with several studies suggests that incorporating SA into niosomes provided more satisfactory stability for synthesized formulation^32,54,55^. In addition, the cationic SA formulation had more EE% than the standard niosomal formulation in both 4 °C and 25 °C, which can be attributed to more interaction of loaded contents with a positive membrane surface. It is proven that zeta potential, either below − 30 mV or above + 30 mV, is considered an acceptable indicator of long-term stability for a charged nano-formulation^56^. The more satisfactory stability of the synthesized cationic formulations (compared with non-cationic formulation) confirms the effectiveness of this range of surface electrical-charge in stabilization of niosomal formulation. However, the results of a study indicated that the PDI, as a significant stability index, increased with the increase of SA concentration in niosomal formulation during the storage period^57^. Therefore, illuminating the exact amount of cationic lipids, including SA, in niosomal formulation and determining an optimizing formulation combined with other niosomal component (cholesterol, Span, Tween) should be considered for developing an optimizing formulation.

Several previous reports have demonstrated that VDDS can enhance the efficacy of the loaded compounds while reducing their toxicity, altering the pharmacokinetics index of the loaded drugs^58–60^. Our findings validated these observations and revealed a reduction in the cytotoxic effect of QCT against the L929 cell line through encapsulation within a niosomal formulation. Furthermore, we observed that cationic niosomes exhibited a more significant reduction in QCT toxicity than standard niosomal formulation, suggesting that including SA in the niosomal composition is an ideal strategy for minimizing undesirable drug side effects in topical administration route. Additionally, our finding aligns with those of previous studies, where SA liposome had no cytotoxic effects in normal human cells such as Human Embryonic Kidney (HEK) 293 and peripheral blood mononuclear cells (PBMCs) at therapeutic concentrations^53,61,62^. These outcomes suggest that optimized formulation with SA could be applied for biomedical applications without causing cytotoxic effects on human erythrocytes and normal cell lines. However, another study reported that increased SA density in liposomal formulation could provoke harmful effects on human RBCs, suggesting that SA minimal hemolytic activity should be considered for intravenous route administration^63^. Therefore, further experiments are warranted in vivo/ex vivo models before developing SA formulations for clinical trials involving various administration routes.

## Conclusion

Based on the results, niosomal DDS could enhance the effective dose of loaded QCT, which can be proposed as a novel anti-*P. aeruginosa* agent. This study revealed that surface modification via incorporating cationic lipid into niosomal formulation could improve the anti-bacterial and anti-biofilm efficacy of QCT-niosomes. The optimized formulation (QCT-SA-niosome) was also more biocompatible compared to the standard formulation (QCT-niosome). Nonetheless, QCT-SA-niosome mediates anti-*P. aeruginosa* of QCT activity by targeting the negatively charged bacterial surfaces. Overall, QCT-SA-niosome is a promising agent in dealing with deadly *P. aeruginosa* infections in the healthcare system, indicating that cationic niosomes can be applied as effective drug carriers in medical applications.

## Data Availability

The datasets used and analyzed during the current study are available from the corresponding author upon reasonable request.
